# Night syncope: a case report

**DOI:** 10.1002/ccr3.757

**Published:** 2016-12-26

**Authors:** Léopold Loumaye, Dominique Blommaert

**Affiliations:** ^1^ CardiologyClinique Universitaire UCL Mont‐GodinneRue Dr G Therasse 1Yvoir5530Belgium

**Keywords:** Defibrillator, polymorphic ventricular tachycardia, sudden death, syncope

## Abstract

“Idiopathic” ventricular fibrillations are rare and are estimated between 5% and 10% of survivors of hospital cardiac arrest, for at least some of them have as origin, a trigger in the Purkinje fibers. Interventional therapy could be an effective, long‐lasting solution for these recurrent malignant arrhythmias and should be considered.

## Introduction

Syncopes remain a diagnostic challenge as well, as an economic one. Indeed, syncopes account for 1% of the emergency consultations and frequently lead to a hospitalization and various investigations 1, 7. The case of the patient described hereafter shows the importance of the anamnesis in an original night case of syncope which final diagnosis was posed only 12 years after the first episode and allows an efficient treatment of her ventricular arrhythmias.

## Clinical Case

In 2001, a 38‐year‐old patient was brought by ambulance to the emergency unit of our university hospital following a syncope during her sleep (04 h 30 min in the morning). Her husband reported a snoring breathing accompanied by an ocular revulsion for a few seconds. He did not describe convulsions or loss of urines but an episode of vomiting during recovery. In addition, the patient did not mention recent trauma, alcohol abuse, drug intake, or recent modification in her treatment (contraceptive pill). In her antecedents, we noticed a cholecystectomy 6 months earlier and a sister with angor as family antecedents. The neurological examination was normal, the patient was conscious and had a fast recovery, and the cardiopulmonary and abdominal examinations were normal. At admission, the ECG revealed a sinus tachycardia at 101/min and no other abnormalities; in particular, the corrected QT duration was calculated at 438 msec. A radiography of the chest and a cerebral scanner were also performed. Both were found to be normal. The EEG was abnormal and suspect of epilepsy (the layout present important anomalies. The rhythm was slightly slowed down without asymmetry. The slow component of the layout was significantly raised, with a prevalence on the band theta, of the right hemisphere).

The results of the blood test were normal, in particular the serum electrolytes (magnesium at 1.6 mg/dL for a normal range between 1.5 and 2.5), except a troponin in a gray area at 1.7 ng/mL (negative <0.4; positive >2.3) and a potassium a little bit low at 3.6 mEq/L (nL 3.7–5.3).

A transthoracic cardiac echography was performed during her stay in the emergency room: It showed a light mitral insufficiency (1/4). A left ventricular function slightly decreased (45–50%). The left ventricle was not dilated and we saw few anomalies of contraction in the inferior segment: light tricuspid insufficiency, discrete pulmonary arterial hypertension (systolic PAP estimated at 31 mmHg + central venous pressure), and no pericardial effusion.

The patient was hospitalized in the intensive care unit for monitoring and to continue investigations. Treatment with valproate was initiated considering possible seizure. An ECG was again performed a few hours after admission and demonstrated T wave who was getting negative in DI, aVL, V5, and V6.

As troponin was slightly elevated, the suspicion of arrhythmia, and a slightly decreased function, a coronary angiography was performed which demonstrated normal coronary arteries and an inferolateral hypokinesia. During the second injection, a ventricular tachycardia started causing a syncope of the patient. It was reduced by external electrical cardioversion. Noteworthy, ventricular tachycardia phenomenon during injection of the right coronary artery is not uncommon and is aspecific.

Immediately after the angiography, the patient underwent an electrophysiological study, which after the third stimulation triggered a ventricular fibrillation episode, which resolved spontaneously. This kind of arrhythmia is also considered as a nonspecific response.

Furthermore, a cardiac MRI was performed and was found to be negative.

At this stage, it was decided collegially (consultation between neurologists, intensivists, and cardiologists) to implant a dual‐chamber defibrillator and the patient was discharged from the hospital a few days later. A follow‐up in neurology was scheduled.

The reasons that led to the implantation of a defibrillator was (i) dynamic ECG abnormalities, (ii) the anamnesis of the husband suggesting an history of cardiac arrest, and (iii) the assumption made by the neurologist of a neurocardiology mixed anomaly with brain damage generating ventricular arrhythmia or a channelopathy affecting both organs (e.g., a mutation of the KCNQ1 gene 8, 9).

Since the implantation in 2001 until 2012, the patient presented 32 episodes of polymorphic not sustained ventricular tachycardia (NSVT), two episodes of polymorphic sustained ventricular tachycardia (SVT) treated by internal defibrillation, and one episode of ventricular fibrillation (VF) also defibrillated in 2003. This last episode was preceded by a bigeminy ectopic ventricular beats (EVBs) of the right ventricle outflow tract. Sotalol therapy was initiated and a base frequency of 80/min was programmed to avoid that EVB. In 2008, the neurologist stopped the valproate because of reassuring EEG and the evidence of recurrent arrhythmias. A 24‐h EEG was also performed 3 weeks after valproate discontinuation and was normal.

**Figure 1:**
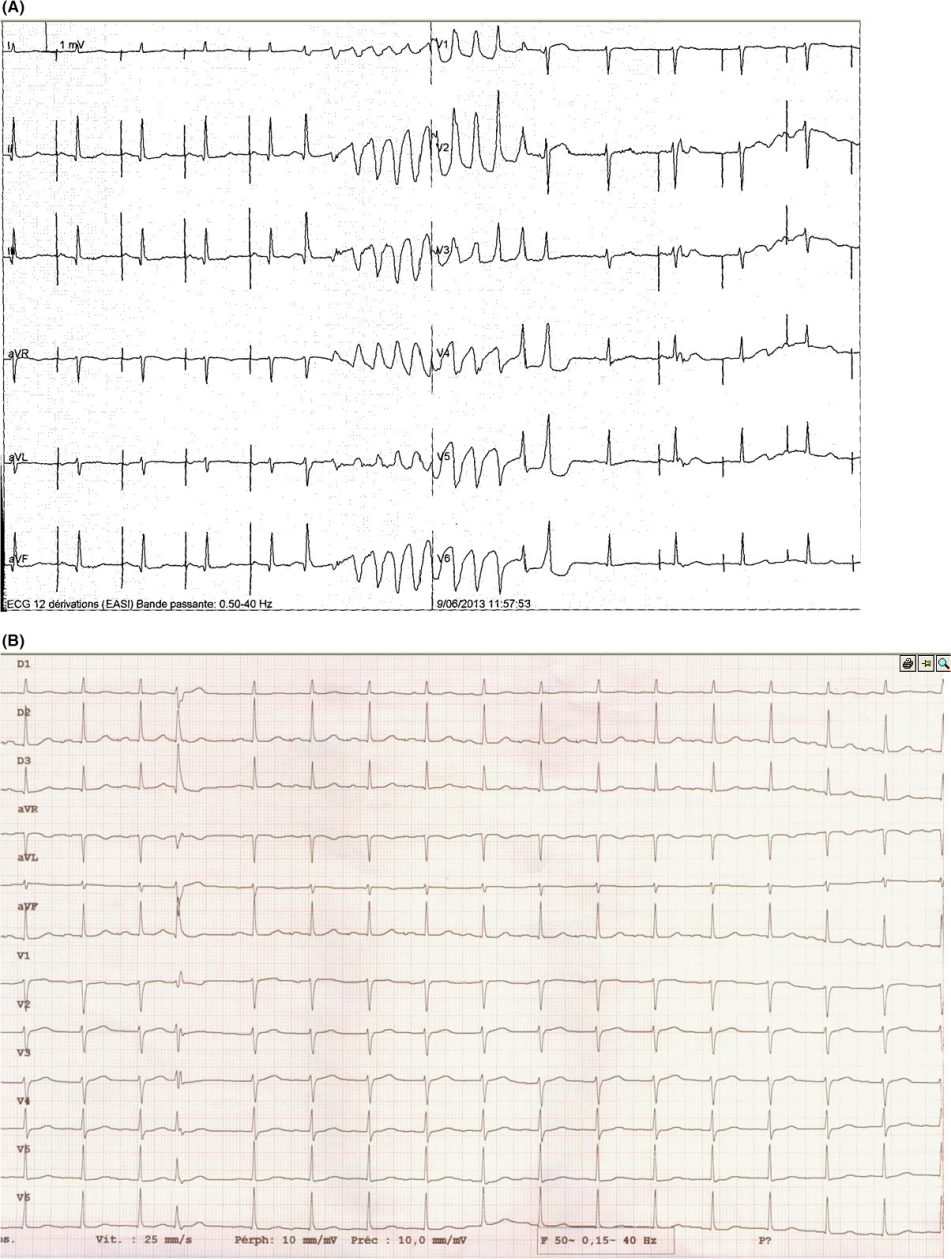
(A) Example of a short episode of malignant arrhythmia in our patient. (B) Type of EVB (12‐lead ECG) inducing arrhythmia.

In June 2013, the patient was again admitted in the emergency room following an internal electric shock. The defibrillator interrogation revealed five polymorphic NSVT in a week, an episode of VF which resolved spontaneously, and a VF treated by an internal electric shock. All these episodes occur after a right delay EVB. The patient was again hospitalized in our cardiology unit during which we recorded for the first time these EVBs with a 12‐lead ECG (Fig. 1).

Following the recording of these malignant arrhythmias during the electrical storm and the initiating premature EVB in 12‐lead ECG, we proposed a diagnostic hypothesis based on the work of Pr. Haïssaguerre 2, 3. His hypothesis is that Purkinje fibers can be the originator of malignant arrhythmias such as “idiopathic” ventricular fibrillation on healthy heart (normal physical examination, normal ECG, normal stress test, normal coronary angiography, normal left and right ventricular function, and exclusion long QT or Brugada). The origin of the arrhythmia trigger is located during electrophysiology through the nearest morphology of the triggering EVB. This hypothesis is based on a 16 patient study in which the origin of these EVBs was in four cases in the outflow tract of the right ventricle, in four cases located in the right Purkinje fibers, seven in the left ones, and one case in both fibers.

When the site was identified, it was ablated by radiofrequency up to disappearance of the EVB. After monitoring by Holter for 3–5 days, patients were discharged without antiarrhythmic drug. In 13 of these 16 patients, the ablation was successful. During a 32‐month follow‐up by Holter, they show an average of 29 EVBs/day and no recurrence of ventricular fibrillation or syncope.

In November 2013, a new electrophysiological study was performed in our patient: The ablation catheter was put into the left ventricle via transseptal puncture and we create an electro‐anatomical map of the left ventricle and fascicles (his + left anterior and posterior fascicles). Various NSVT appeared when the catheter touched the left fascicles. The morphology of the “mechanical” EVB was very close to the ones recorded during the last hospitalization. In the absence of spontaneous arrhythmia despite isoprenaline, we decided to carry out few applications at the distal part of the anterior left fascicle. The NSVT stopped gradually during radiofrequency shots.

At the end of the procedure, there were no changes in 12‐lead ECG.

Currently, 26 months after the electrophysiological intervention, the patient has no recurrence of sustained ventricular arrhythmia with only sotalol 80 mg 2x/day as treatment.

## Discussion

The diagnosis of idiopathic ventricular fibrillation is an exclusion diagnosis. Indeed, we have to exclude on the one hand structural heart disease such as arrhythmogenic right ventricular dysplasia, ischemic cardiomyopathies, cardiomyopathies, and valvular cardiomyopathies and on the other hand the purely rhythmic pathologies such as long QT syndrome, Brugada syndromes, or catecholaminergic polymorphic ventricular tachycardia 4, 5.

These “idiopathic” ventricular fibrillations are rare and are estimated between 5% and 10% of survivors of hospital cardiac arrest 6 and most likely, for at least some of them have as origin, a trigger in the Purkinje fibers as postulated by Pr. Haïssaguerre. Our case therefore raised several important points. First, it emphasizes the importance of the history and especially the anamnesis of the witnesses of the loss of consciousness in the management of syncope. Second, the decision to implant a defibrillator without clear arguments but based on an interdisciplinary consensus justified by many effective treatments the patient received who saved her life. Finally, interventional therapy could be an effective, long‐lasting solution for these recurrent malignant arrhythmias and should be considered.

## Conclusion

In conclusion, the patient received an implantable defibrillator in 2001 based on a history of night syncope and multiple ventricular arrhythmias (VT, torsade de pointes, and VF) without precise origin. This was associated with a reduction of many of these arrhythmias including for VF. Twelve years later, after multiple therapies delivered by the defibrillator, the triggering factor of these arrhythmias was identified as EVBs from the anterior left fascicle and treated by radiofrequency.

## Authorship

LL: is the main author, he writes this paper and makes a review of the litterature of the different causes of malignant arrythmias. DB: is the doctor in charge of the patient, he has the idea to treat this patient by radiofrequency and he makes the review of this article.

## Conflict of Interest

None declared.
